# Supplementary material for: Human ACE2 transgenic pigs are susceptible to SARS-CoV-2 and develop COVID-19-like disease
Source: Nat Commun. 2025 Jan 17;16:766. doi: 10.1038/s41467-024-54615-1 (PMC11742018; doi:10.1038/s41467-024-54615-1)
Supplement: Supplementary file 1 — Supplementary Information [file 41467_2024_54615_MOESM1_ESM.pdf]

Supplemental Information

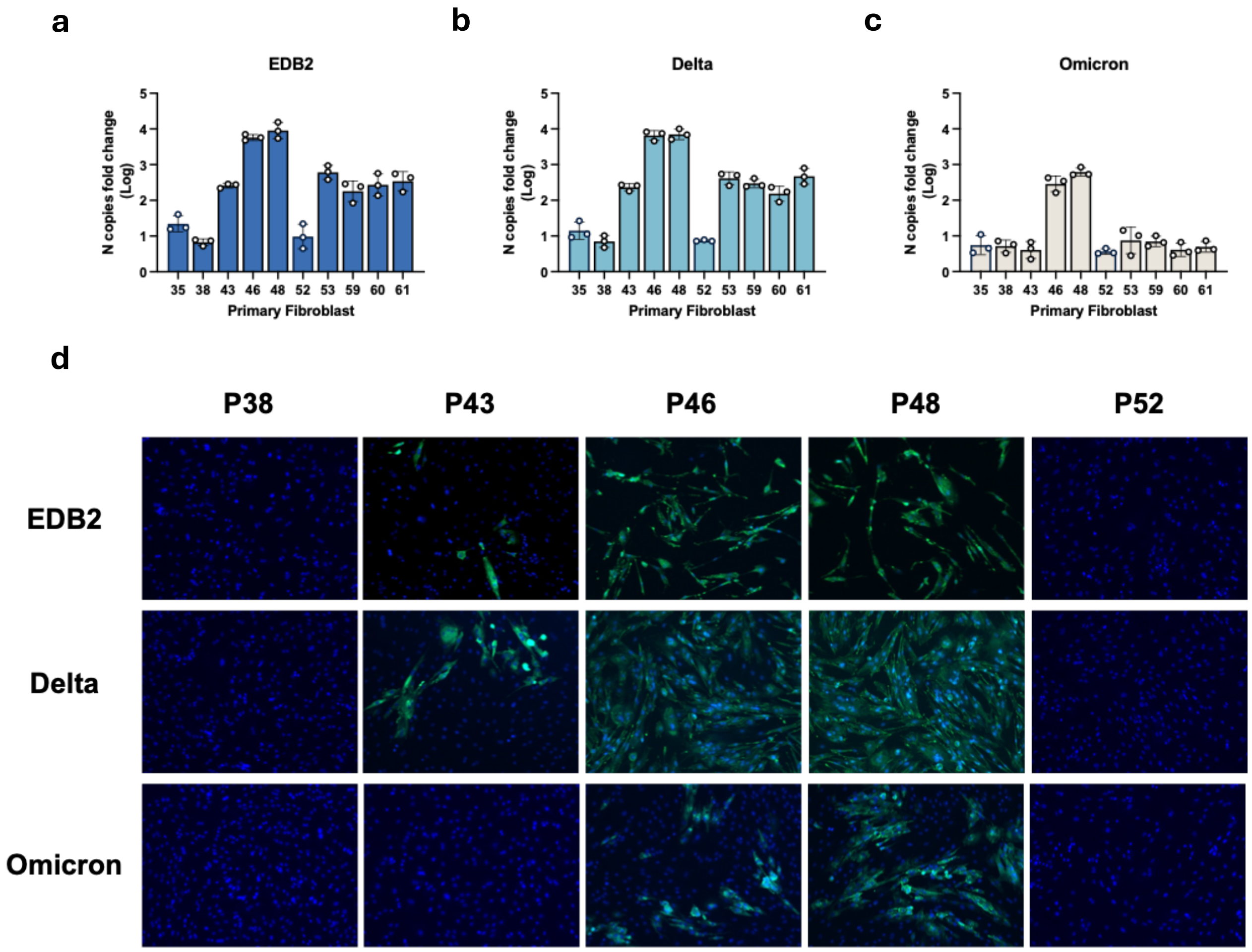

Supplemental figure 1. Replication of delta and omicron variants in primary fibroblast cells from hACE2 transgenic pigs. Total RNA was extracted from supernatant 48 HPI and relative viral genome levels determined using RT-qPCR for (a) EDB2, (b) Delta and (c) Omicron following infection of primary fibroblast cells from transgenic pigs. Data are mean ± S.D. from three independent experiments, each using two technical replicates. Source data are provided as a Source Data file. (d) Susceptibility of selected cells was confirmed using immunofluorescent staining. Representative images were shown from three independent experiments. SARS-CoV-2 N protein = green. DAPI = blue.

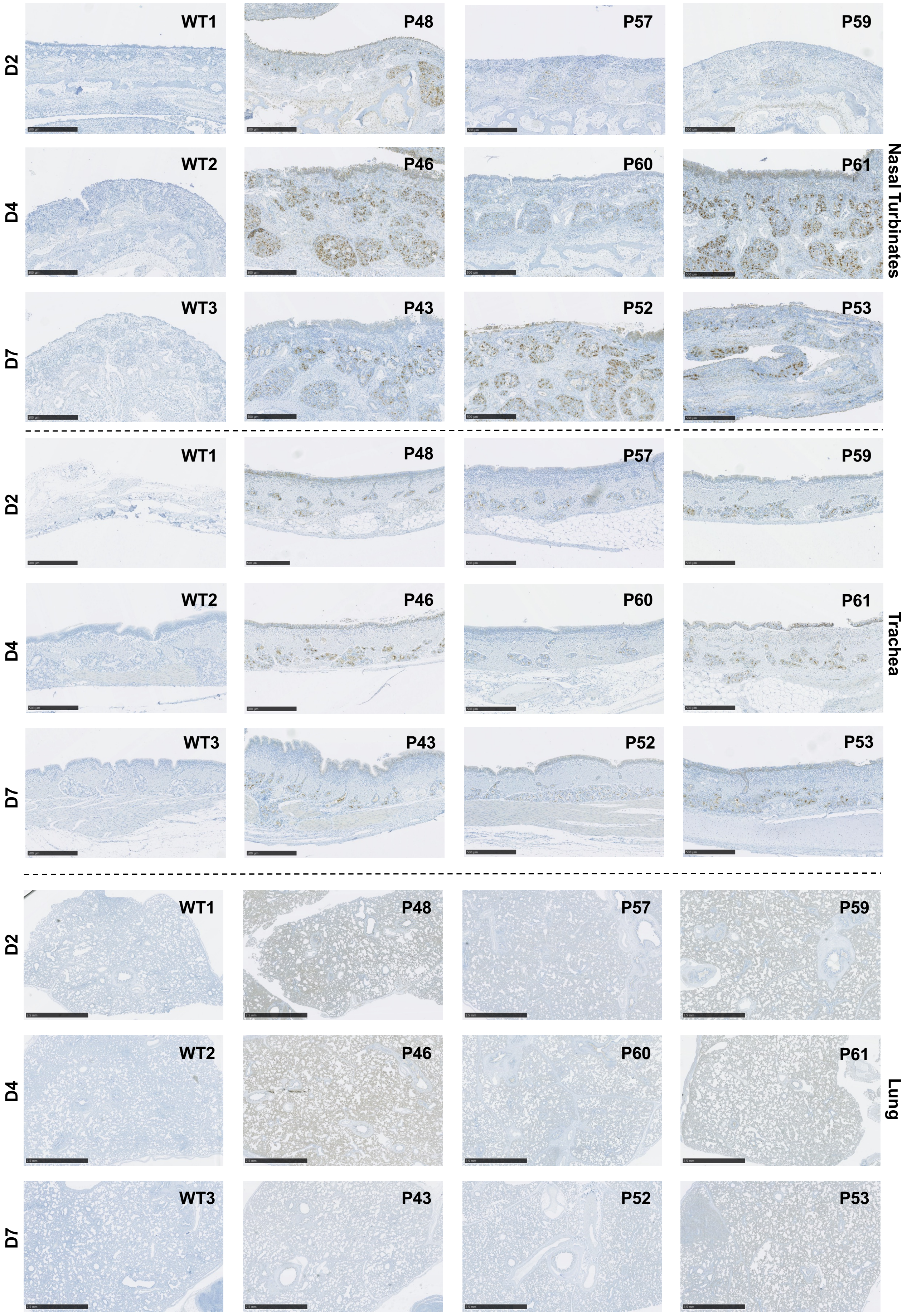
Nasal Turbinates
Trachea
Lung
D2
D4
D7
WT1
P48
P57
P59
WT2
P46
P60
P61
WT3
P43
P52
P53

Supplemental figure 2. Representative images of hACE2 staining in the upper and lower respiratory tract. Brown staining indicates hACE2 expression against a blue haematoxylin counterstain. hACE2 expression is mainly associated with epithelial cells with variable levels of expression between individual pigs. hACE2 expression occurs in apical epithelial cells and cells associated with mucus glands in nasal turbinates and trachea, while expression in the lungs is associated with alveolar structures. No staining is observed in tissues from WT control pigs. 500 µm scale bars are shown for nasal turbinates and trachea (5x magnification), 2.5 mm scale bars are shown for lung tissue (1x magnification).

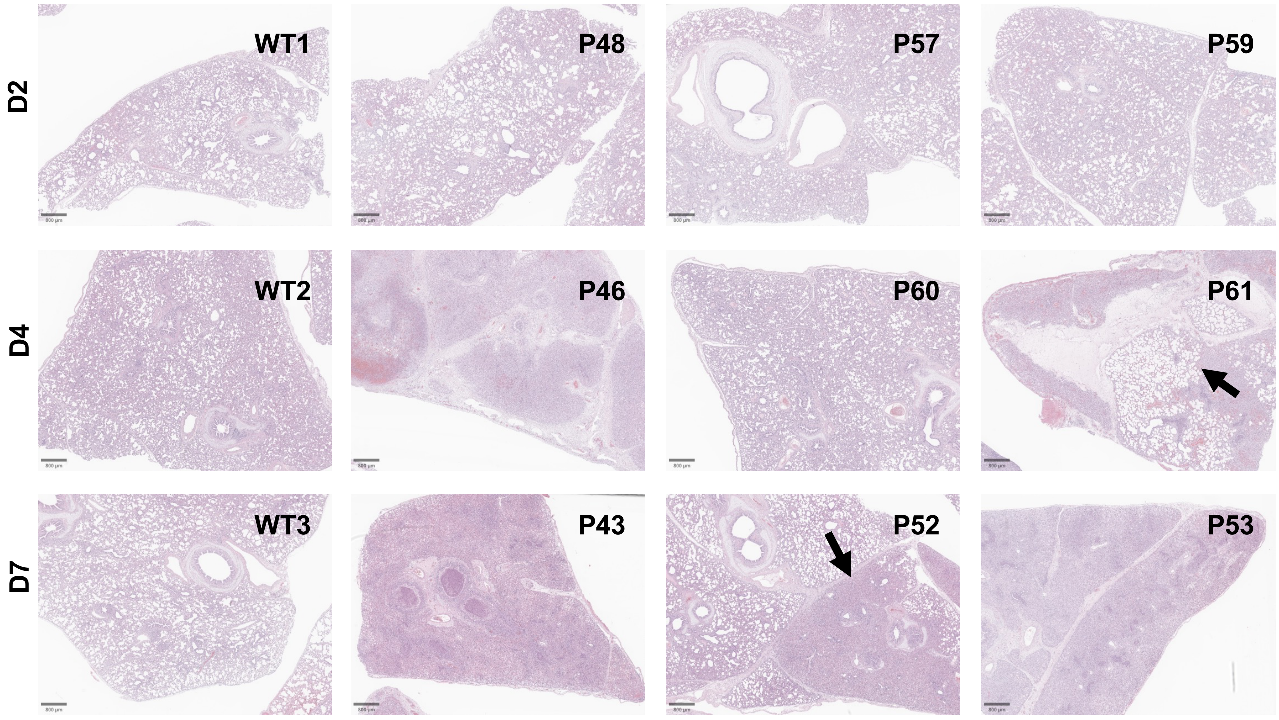

Supplemental figure 3. Histopathology in lungs of hACE2 infected pigs compared to WT controls. Sections were stained with haematoxylin and eosin for histology analysis. Photomicrographs of lung sections show extensive inflammation and hepatization of lungs from four DPI. Arrows indicate demarcation between regions of lung hepatization within the same tissue samples (P61, P52) suggest asymmetrical delivery of viral inoculum. Scale bars are 800µm (1.2x magnification).

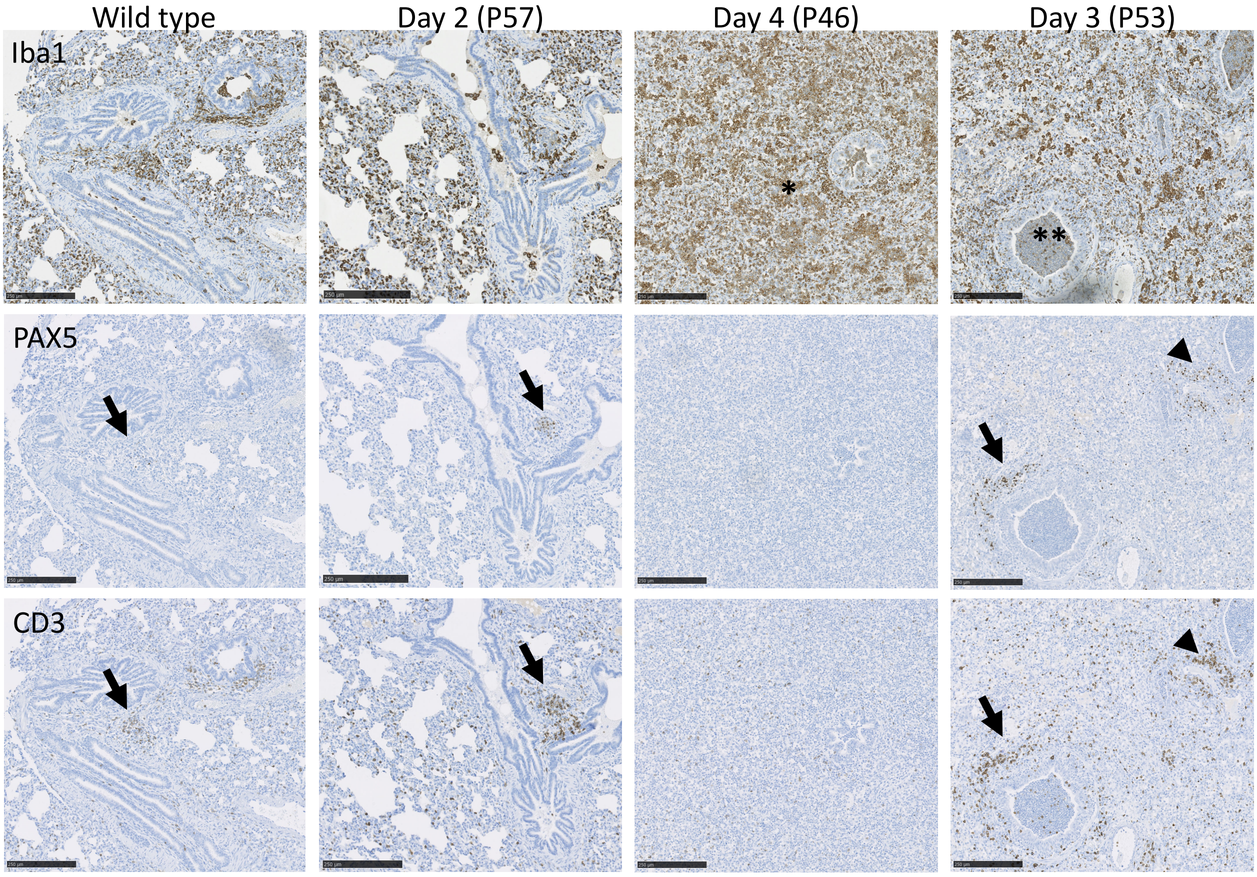

Supplemental figure 4. Inflammatory cell populations following SARS-CoV-2 infection. Wild type lungs contained very occasional peribronchiolar (arrow) B- and T-cell lymphoid aggregates (PAX5 & CD3 respectively) along with interstitial and alveolar macrophages (Iba1); similar to uninfected animals (data not shown). At day 2 following infection of hACE2 animals there was no observable difference in macrophage and lymphoid populations. In the context of significant neutrophil and macrophage predominant inflammation at day 4 sparse, dispersed lymphocytes were identified but only very sparse lymphoid aggregates. Expanded alveolar (*) and intra-bronchiolar (**) macrophage populations are present at day 4 and 7. Prominent peribronchiolar and perivascular (arrowhead) B and T cell populations present at day 7. Qualitative description only due to variation in lung inflation at time of tissue retrieval. Representative images shown (n=3 hACE2 animals/time point). Scale bars = 250μm (10x magnification).

Dam	Pig	sex	Proviral (Ct)
5386	P1	m	20.88
5386	P2	m	21.07
5386	P3	m	22.79
5386	P4	m	22.34
5386	P5	f	22.18
5386	P6	f	23.88
5386	P7	f	20.86
5386	P8	f	22.16
5386	P9	f	21.85
5386	P10	f	21.5
5386	P11	f	30.76
5386	P12	f	22.62
5386	P13	f	19.73
5258	P14	m	22.85
5258	P15	m	22.38
5258	P16	m	25.46
5258	P17	m	21.23
5258	P18	m	22.05
5258	P19	m	23.62
5258	P20	f	21.2
5258	P21	f	21.95
5258	P22	f	21.63
5258	P23	f	ND
5258	P24	f	27.15
5258	P25	f	27.8
5258	P26	f	21.37
5258	P27	f	27.1
5305	P28	m	29.75
5305	P29	m	27.81
5305	P30	m	22.36
5305	P31	f	ND
5305	P32	f	ND

Supplemental table 1. Relative levels of lentivirus integrated into the genome of F0 transgenic pigs was determined using the Lenti-X Provirus Quantitation Kit. For each pig, the surrogate dam is indicated and the sex (m = male, f = female). ND indicates not detected. Pigs selected for breeding of F1 cohort are highlighted in green (females) and blue (males). Grey text indicates pigs that were culled due to an outbreak of Staphylococcus hyicus.

Parentage	Cohort	Sex	Pig	Relative hACE2 expression
P2xP7	1	F	P48	31.12
P2xP13	1	F	P57	25.99
P2xP13	1	F	P59	39.95
P2xP7	2	F	P46	51.27
P2xP13	2	F	P60	27.28
P2xP13	2	F	P61	53.82
P2xP7	3	F	P43	25.46
P2xP13	3	M	P52	19.56
P2xP13	3	M	P53	19.16
P2xP13	UN	F	P56	15.45
P2xP13	UN	F	P58	15.45
P2xP7	UN	M	P38	0.02
P2xP7	UN	M	P35	0.02
P2xP13	X	M	P54	15.35
P2xP13	X	M	P51	13.18
P2xP7	X	M	P39	8.88
P2xP7	X	F	P44	7.46
P2xP7	X	M	P37	7.21
P2xP13	X	M	P55	6.15
P4xP26	X	F	P62	2.39
P2xP7	X	F	P50	2.23
P2xP7	X	F	P45	1.71
P2xP7	X	F	P47	1.49
P2xP7	X	M	P34	1.29
P2xP7	X	M	P36	1.06
P2xP7	X	M	P33	1
P2xP7	X	F	P42	0.23
P2xP7	X	F	P49	0.03
P2xP7	X	M	P40	0.03

Supplemental table 2. Relative expression of Human ACE2 transcript in ear tissue from F1 pigs. Total RNA was extracted from ear tags and hACE2 transcript levels determined by RT-qPCR. Levels were normalised to GAPDH and relative levels determined against P33. P33 was chosen as an arbitrary base line expression level for comparison with other pigs. F0 parentage is shown and selection for cohort for challenge study indicated. UN indicates pigs selected for uninfected controls. X indicates pigs not used in challenge study. Relative expression is colour formatted to indicate high levels in green and low levels in red.

Cohort	Pig ID	Clinical observations
**Cohort 1 (2 DPI)**	WT1	No abnormal clinical signs observed at any time
	P48	24 hours: Occasional cough<br>48 hours: Occasional cough
	P57	No abnormal clinical signs observed at any time
	P59	No abnormal clinical signs observed at any time
**Cohort 2 (4 DPI)**	WT2	No abnormal clinical signs observed at any time
	P46	24 hours: Mild increase in respiratory effort; occasional cough<br>72 hours: Reduced responsiveness, mild increase in respiratory effort, occasional cough<br>84 hours: Reduced responsiveness, mild increase in respiratory effort<br>96 hours: Lethargic, reluctant to stand; extended intermittent periods of coughing; laboured respiration, nasal discharge
	P60	72 hours: Reduced responsiveness, mild increase in respiratory effort<br>84 hours: Reduced responsiveness, mild increase in respiratory effort, occasional cough<br>96 hours: Laboured respiration, nasal discharge
	P61	24 hours: Mild increase in respiratory effort; occasional cough<br>48 hours: Occasional cough<br>72 hours: Reduced responsiveness; laboured respiration, nasal discharge<br>84 hours: Reduced responsiveness, mild increase in respiratory effort<br>96 hours: Moderate increase in respiratory effort
**Cohort 3 (7 DPI)**	WT3	No abnormal clinical signs observed at any time
	P43	72 hours: Reduced responsiveness, mild increase in respiratory effort, occasional cough<br>84 hours: Reduced responsiveness, mild increase in respiratory effort, occasional cough<br>96 hours: Moderate increase in respiratory effort<br>108 hours: No abnormal clinical signs observed<br>120 hours: No abnormal clinical signs observed<br>144 hours: Mild increase in respiratory effort; occasional cough<br>156 hours: Mild increase in respiratory effort
	P52	72 hours: Reduced responsiveness, mild increase in respiratory effort<br>84 hours: Reduced responsiveness, laborious respiration and nasal discharge<br>96 hours: Moderate increase in respiratory effort<br>108 hours: No abnormal clinical signs observed<br>120 hours: Mild increase in respiratory effort<br>132 hours: Occasional sneezing<br>144 hours: Mild increase in respiratory effort; occasional cough and sneezing<br>156 hours: Mild increase in respiratory effort, occasional cough and sneezing
	P53	48 hours: Occasional cough<br>72 hours: Reduced responsiveness, mild increase in respiratory effort<br>72 hours: Reduced responsiveness, mild increase in respiratory effort, occasional cough<br>96 hours: Moderate increase in respiratory effort<br>108 hours: Occasional cough<br>120 hours: Mild increase in respiratory effort; occasional cough ; nasal discharge<br>132 hours: Occasional cough<br>144 hours: Reduced responsiveness, mild increase in respiratory effort, occasional cough and sneezing<br>156 hours: Mild increase in respiratory effort, occasional cough and sneezing

Supplemental table 3. Summary of clinical assessments for each pig, arranged by cohort (cohort 1 culled two DPI, cohort 2 culled four DPI, cohort three culled seven DPI). Design of clinical assessment scoring system is described in materials and methods.

		Percentage
Day 2	WT1	0
	P48	32
	P57	25
	P59	28
Day 4	WT2	0
	P46	87
	P60	25
	P61	92
Day 7	WT3	18
	P43	74
	P52	14
	P53	55

Supplemental table 4. Whole lung lesion assessment. An estimated percentage of the lung with grossly visible pneumonia was recorded for each pig based on a previously described scoring system. Each lung lobe was assigned a number to reflect the approximate volume or percentage of the entire lung represented by that lobe. Ten possible points each were assigned to the right cranial lobe, right middle lobe, cranial part of the left cranial lobe, and caudal part of the left cranial lobe. The accessory lobe was assigned 5 points. The right and left caudal lobes were each assigned 27.5 points to reach a total of 100 points. The total for all the lobes was an estimate of the percentage of the entire visible pneumonia.

		Alveolar injury		Suppurative Pneumonia		Parenchymal Necrosis		Pleuritis		Bronchiolar necrosis		Bronchiolar inflammation	
		1	2	1	2	1	2	1	2	1	2	1	2
Day 2	WT1												
	P48			■	■		■					■	
	P57		■		■						■	■	■
	P59						■						
Day 4	WT2												
	P46	■	■	■	■	■	■	■	■	■	■	■	■
	P60		■			■							
	P61	■	■	■	■		■	■	■	■	■	■	■
Day 7	WT3											■	■
	P43	■	■	■	■	■	■	■		■	■	■	■
	P52	■	■	■			■						
	P53	■	■	■	■		■				■	■	■

Supplemental table 5. Scoring of inflammatory pathology in lung tissue. Lung histology was assessed blind by two pathologists (1 and 2) based on a binary presence or absence scoring system. Pigs are group according to challenge cohort. Red indicates presence of pathology.
