# Supplementary material for: Human ACE2 transgenic pigs are susceptible to SARS-CoV-2 and develop COVID-19-like disease
Source: Nat Commun. 2025 Jan 17;16:766. doi: 10.1038/s41467-024-54615-1 (PMC11742018; doi:10.1038/s41467-024-54615-1)
Supplement: Supplementary file 3 — Reporting Summary [file 41467_2024_54615_MOESM3_ESM.pdf]

nature portfolio

Corresponding author(s): David Griffiths and Finn Grey

Last updated by author(s): 19-4-2024

Reporting Summary

Nature Portfolio wishes to improve the reproducibility of the work that we publish. This form provides structure for consistency and transparency in reporting. For further information on Nature Portfolio policies, see our Editorial Policies and the Editorial Policy Checklist.

Statistics

For all statistical analyses, confirm that the following items are present in the figure legend, table legend, main text, or Methods section.

n/a	Confirmed	
☐	☒	The exact sample size (n) for each experimental group/condition, given as a discrete number and unit of measurement
☐	☒	A statement on whether measurements were taken from distinct samples or whether the same sample was measured repeatedly
☒	☐	The statistical test(s) used AND whether they are one- or two-sided<br>*Only common tests should be described solely by name; describe more complex techniques in the Methods section.*
☒	☐	A description of all covariates tested
☒	☐	A description of any assumptions or corrections, such as tests of normality and adjustment for multiple comparisons
☐	☒	A full description of the statistical parameters including central tendency (e.g. means) or other basic estimates (e.g. regression coefficient) AND variation (e.g. standard deviation) or associated estimates of uncertainty (e.g. confidence intervals)
☒	☐	For null hypothesis testing, the test statistic (e.g. F, t, r) with confidence intervals, effect sizes, degrees of freedom and P value noted<br>*Give P values as exact values whenever suitable.*
☒	☐	For Bayesian analysis, information on the choice of priors and Markov chain Monte Carlo settings
☒	☐	For hierarchical and complex designs, identification of the appropriate level for tests and full reporting of outcomes
☐	☒	Estimates of effect sizes (e.g. Cohen's d, Pearson's r), indicating how they were calculated

Our web collection on statistics for biologists contains articles on many of the points above.

Software and code

Policy information about availability of computer code

Data collection	No software was used.
Data analysis	Prism version 10.2.1 and excel 16.82 was used for generation of graphs. NDP view 2 was used for image generation for histology. The schematic for generation of transgenic pigs was made in biorender. All other figures were generated in Powerpoint version 16.81.

For manuscripts utilizing custom algorithms or software that are central to the research but not yet described in published literature, software must be made available to editors and reviewers. We strongly encourage code deposition in a community repository (e.g. GitHub). See the Nature Portfolio guidelines for submitting code & software for further information.

Data

Policy information about availability of data

All manuscripts must include a data availability statement. This statement should provide the following information, where applicable:

- Accession codes, unique identifiers, or web links for publicly available datasets
- A description of any restrictions on data availability
- For clinical datasets or third party data, please ensure that the statement adheres to our policy

All data supporting the findings of this study are available within the paper and its Supplementary Information.

Research involving human participants, their data, or biological material

Policy information about studies with human participants or human data. See also policy information about sex, gender (identity/presentation), and sexual orientation and race, ethnicity and racism.

Reporting on sex and gender	*Use the terms sex (biological attribute) and gender (shaped by social and cultural circumstances) carefully in order to avoid confusing both terms. Indicate if findings apply to only one sex or gender; describe whether sex and gender were considered in study design; whether sex and/or gender was determined based on self-reporting or assigned and methods used.*<br>*Provide in the source data disaggregated sex and gender data, where this information has been collected, and if consent has been obtained for sharing of individual-level data; provide overall numbers in this Reporting Summary. Please state if this information has not been collected.*<br>*Report sex- and gender-based analyses where performed, justify reasons for lack of sex- and gender-based analysis.*
Reporting on race, ethnicity, or other socially relevant groupings	*Please specify the socially constructed or socially relevant categorization variable(s) used in your manuscript and explain why they were used. Please note that such variables should not be used as proxies for other socially constructed/relevant variables (for example, race or ethnicity should not be used as a proxy for socioeconomic status).*<br>*Provide clear definitions of the relevant terms used, how they were provided (by the participants/respondents, the researchers, or third parties), and the method(s) used to classify people into the different categories (e.g. self-report, census or administrative data, social media data, etc.)*<br>*Please provide details about how you controlled for confounding variables in your analyses.*
Population characteristics	*Describe the covariate-relevant population characteristics of the human research participants (e.g. age, genotypic information, past and current diagnosis and treatment categories). If you filled out the behavioural & social sciences study design questions and have nothing to add here, write "See above."*
Recruitment	*Describe how participants were recruited. Outline any potential self-selection bias or other biases that may be present and how these are likely to impact results.*
Ethics oversight	*Identify the organization(s) that approved the study protocol.*

Note that full information on the approval of the study protocol must also be provided in the manuscript.

Field-specific reporting

Please select the one below that is the best fit for your research. If you are not sure, read the appropriate sections before making your selection.

☒ Life sciences ☐ Behavioural & social sciences ☐ Ecological, evolutionary & environmental sciences

For a reference copy of the document with all sections, see nature.com/documents/nr-reporting-summary-flat.pdf

Life sciences study design

All studies must disclose on these points even when the disclosure is negative.

Sample size	Sample size was based on the maximum capacity of the CL3 containment rooms for large animal challenge studies at the Moredun Research Institute. Maximum capacity was determined as 12 for pigs aged between 6-10 weeks. Based on this, the study design included 3 cohorts of pigs each including 3 transgenic pigs and 1 non-transgenic control pig, allowing for 3 time points durng the study, days 2, 4 and 7 post infection.
Data exclusions	No data was excluded from the study
Replication	RNA was isolated independently from cells generated from pigs to allow replication of hACE2 expression levels. For TCID50 experiments, each sample was divided into two, which were separately subjected to 10-fold dilutions (10-1 to 10-6) in serum-free DMEM. For each sample and dilution, eight replicate wells were used for infection.
Randomization	Initial design of challenge studies were based on hACE2 expression levels from ear biopsies, with each cohort containing a pig expressing high, medium and low levels of hACE2. However cohorts were reassessed as the challenge study progressed, with pigs showing greater degrees of clinical signs selected at early time points to avoid the requirement for emergency postmortem procedures at CL3 containment. We acknowledge this will introduce bias to the study, but was deemed necessary for both safety and animal welfare considerations.
Blinding	Pathology scores were generated by two pathologists, who were blinded to each others scores and the sample identifier. Scores were compared and reconciled following independent scoring.

Reporting for specific materials, systems and methods

We require information from authors about some types of materials, experimental systems and methods used in many studies. Here, indicate whether each material, system or method listed is relevant to your study. If you are not sure if a list item applies to your research, read the appropriate section before selecting a response.

Materials & experimental systems

n/a	Involved in the study
☐	☒ Antibodies
☐	☒ Eukaryotic cell lines
☒	☐ Palaeontology and archaeology
☐	☒ Animals and other organisms
☒	☐ Clinical data
☒	☐ Dual use research of concern
☒	☐ Plants

Methods

n/a	Involved in the study
☒	☐ ChIP-seq
☒	☐ Flow cytometry
☒	☐ MRI-based neuroimaging

Antibodies

Antibodies used	hACE2 - rabbit monoclonal antibody (ab108209; ABCAM; 1:150). SARS-CoV-2 Nucleocapsid protein - mouse monoclonal (B46F; Invitrogen; 1:100). For immune cell staining, rabbit anti IBa1 (019-19741; Wako; 1 �g/ml), mouse monoclonal PAX5 (AB_398182; Becton and Dickinson; 5 �g/ml) and mouse monoclonal CD3 (NCL-L-CD3-565; Novacastra; 0.2��g/ml) were used for macrophage, B and T cell staining respectively.
Validation	Known hACE2 and SARS-CoV-2 positive and negative sections were used to validate the hACE2 and SARS-CoV-2 antibodies ABCAM website states the antibody has been confirmed for specificity using hACE2 KO cells. The antibody has been cited 19 times. The SARS-CoV-2 antibody has been cited 16 times according to the product website. Pig brain was used as control material for Iba1, and pig kidney/heart was used as initial ACE2 controls.

Eukaryotic cell lines

Policy information about cell lines and Sex and Gender in Research

Cell line source(s)	Newborn Swine Kidney cells (NSK; RRID: CVCL_8378) expressing human ACE2 were generated through transduction with pLenti-CMV-hACE2-hygro. Lenti-X 293T cells were commercially sourced from Takara.
Authentication	RNAseq data confirmed NSK cells are of swine origin.
Mycoplasma contamination	All cell lines tested negative for mycoplasma.
Commonly misidentified lines (See ICLAC register)	*Name any commonly misidentified cell lines used in the study and provide a rationale for their use.*

Animals and other research organisms

Policy information about studies involving animals; ARRIVE guidelines recommended for reporting animal research, and Sex and Gender in Research

Laboratory animals	Large-White/Landrace pigs were used for generation of transgenic pigs. Eight week old pigs were used in the challenge study
Wild animals	The study did not use wild aniimals.
Reporting on sex	It was not possible to balance genders in the challenge study do to the sex ratio of the pigs with high expression of hACE2.
Field-collected samples	The study did not involve field collected samples.
Ethics oversight	The transgenic pigs were generated under the license PEFC7DB6A "Production, maintenance and analysis of genetically altered livestock" and the procedures approved by the local veterinarian. The challenge study was performed under the Home Office License "Infectious disease in pigs and ruminants", PPL number: PP4132205, Protocol 1, granted on 08-July 2021 and expires on 08 July 26. The infection study was approved by the Moredun Research Institute Animal Welfare and Ethical Review body (AWERB) with internal reference number E33/23 (approval date 18 July 2023). Environmental enrichment was provided and the animal care technicians overlooking the pigs on a regular basis were not the investigators. All animal studies were performed with local and national ethical approval in accordance with the Animal (Scientific Procedures) Act 1986.

Note that full information on the approval of the study protocol must also be provided in the manuscript.

Plants

Seed stocks	*Report on the source of all seed stocks or other plant material used. If applicable, state the seed stock centre and catalogue number. If plant specimens were collected from the field, describe the collection location, date and sampling procedures.*
Novel plant genotypes	*Describe the methods by which all novel plant genotypes were produced. This includes those generated by transgenic approaches, gene editing, chemical/radiation-based mutagenesis and hybridization. For transgenic lines, describe the transformation method, the number of independent lines analyzed and the generation upon which experiments were performed. For gene-edited lines, describe the editor used, the endogenous sequence targeted for editing, the targeting guide RNA sequence (if applicable) and how the editor was applied.*
Authentication	*Describe any authentication procedures for each seed stock used or novel genotype generated. Describe any experiments used to assess the effect of a mutation and, where applicable, how potential secondary effects (e.g. second site T-DNA insertions, mosiacism, off-target gene editing) were examined.*
